# Recent Development of Nano-Materials Used in DNA Biosensors

**DOI:** 10.3390/s90705534

**Published:** 2009-07-14

**Authors:** Kai Xu, Junran Huang, Zunzhong Ye, Yibin Ying, Yanbin Li

**Affiliations:** 1 School of Biosystems Engineering and Food Science, Zhejiang University, Hangzhou, Zhejiang Province, China, 310029; E-Mails: xk021402173@yahoo.com.cn (K.X.); hjr8400@yahoo.com.cn (J.H.); zzye@zju.edu.cn (Z.Y.); 2 Qingdao Institute of Bioenergy and Bioprocess Technology, Chinese Academy of Sciences; Key Laboratory of Biofuels, Chinese Academy of Sciences, Qingdao, Shandong Province, 266101, China; 3 Department of Biological & Agricultural Engineering, University of Arkansas, Fayetteville, AR 72701, USA; E-Mail: yanbinli@uark.edu

**Keywords:** nano-material, DNA biosensor, substrates, signal amplification

## Abstract

As knowledge of the structure and function of nucleic acid molecules has increased, sequence-specific DNA detection has gained increased importance. DNA biosensors based on nucleic acid hybridization have been actively developed because of their specificity, speed, portability, and low cost. Recently, there has been considerable interest in using nano-materials for DNA biosensors. Because of their high surface-to-volume ratios and excellent biological compatibilities, nano-materials could be used to increase the amount of DNA immobilization; moreover, DNA bound to nano-materials can maintain its biological activity. Alternatively, signal amplification by labeling a targeted analyte with nano-materials has also been reported for DNA biosensors in many papers. This review summarizes the applications of various nano-materials for DNA biosensors during past five years. We found that nano-materials of small sizes were advantageous as substrates for DNA attachment or as labels for signal amplification; and use of two or more types of nano-materials in the biosensors could improve their overall quality and to overcome the deficiencies of the individual nano-components. Most current DNA biosensors require the use of polymerase chain reaction (PCR) in their protocols. However, further development of nano-materials with smaller size and/or with improved biological and chemical properties would substantially enhance the accuracy, selectivity and sensitivity of DNA biosensors. Thus, DNA biosensors without PCR amplification may become a reality in the foreseeable future.

## 1. Introduction

With the increased understanding of the structure, organization, sequence and function of nucleic acid molecules, sequence-specific DNA detection has become increasingly important. Detection of specific DNA sequences is needed in many fields: the Human Genome Project is providing massive amounts of genetic information that should revolutionize our understanding and diagnosis of inherited diseases [1]; pathogens responsible for human and animal diseases, bacteria and viruses, are detectable via their unique nucleic acid sequences [2–5]; genetically modified organisms (GMOs) could also be detected via their specific nucleic acid fragments of artificial introduction [6,7]. It also holds enormous potential for the development of new and specific therapeutic procedures, new drug research and development, gene therapy, food technology, environmental sciences, etc [8–12]. Therefore, development of a simple, rapid and user-friendly method for specific DNA sequences testing has become increasingly important to meet these needs.

Conventional methods for the analysis of specific gene sequences are carried out using gel electrophoresis of DNA fragments amplified by the polymerase chain reaction (PCR) using primers that are sequence-specific for the chosen region of DNA. Although it is simple and effective for the detection of PCR products, gel electrophoresis fails to provide any sequence information about the amplified DNA. Furthermore, the use of PCR is limited to laboratories because of its complexity in obtaining proper primers design and the corresponding empirical conditions for consistent amplification [13]. Southern blotting satisfies the requirement for sequence information, but it is not recommended for routine analysis, as it involves several steps [14].

In recent years, DNA biosensors based on nucleic acid hybridization have been vigorously pursued. DNA biosensors are defined as analytical devices incorporating a single-stranded oligonucleotide (probe) intimately associated with or integrated within a physicochemical transducer or transducing microsystem, which may be optical, electrochemical, thermometric, piezoelectric, magnetic or micromechanical. The aim of a DNA biosensor usually is to produce either discrete or continuous measurable signals, which are proportional to the concentration of complementary (target) DNA sequence. Because of their specificity, speed, portability, and low cost, DNA biosensors offer exciting opportunities for sequence-specific DNA detection. However, the concentration of genetic targets is very low in biological samples, making it unsuitable for detection by a DNA biosensor. Therefore, an ultrasensitive method of detecting nucleic acids is clearly essential.

In order to achieve high detection sensitivity, researchers have developed many techniques to enhance the response of DNA biosensors by modifying the sensors with different functional materials. Within the growing and increasingly complex area of nanotechnology, great attention has been paid in recent years to nano-structured materials of different chemical composition, produced as nanoparticles, nanowires or nanotubes. Nano-materials are larger than individual atoms and molecules but are smaller than bulk solids, therefore they obey neither absolute quantum chemistry nor the laws of classical physics and have properties that differ markedly from those expected. There are two major phenomena that are responsible for these differences. First is the high dispersity of nanocrystalline systems [15]. As the size of a crystal reduces, the number of atoms at the surface of the crystal compared to the number of atoms in the crystal itself, increases. The second is called size quantization and arises because the size of nano-materials is comparable to the de Broglie wavelength of its charge carriers [16]. Because of the spatial confinement of the charge carriers, the edge of the valance and conduction bands split into discrete, quantized, electronic levels. These electronic levels are similar to those in atoms and molecules. Because of these two unique phenomena which occur in nano-materials, their properties (electrical, optical, chemical, mechanical, magnetic, etc.) can be selectively controlled by engineering the size, morphology, and composition of the particles. These new substances will have enhanced or entirely different properties from their parent materials.

The nano-materials used in DNA biosensors including nanoparticles, like gold (Au) nanoparticles, Cadmium sulfide (CdS) nanoparticles; nanowires like silicon (Si) nanowires, nanotubes like carbon nanotubes, etc. There are mainly two purposes of using nano-materials in DNA biosensors: as substrates for DNA attachment and as signal amplifiers for hybridization. The aim of this article is to summarize the various nano-materials used in DNA biosensors based on the two purposes in recent years, including the information on applications and future prospects.

## 2. Nano-Material as Substrates for DNA Attachment

As we know, the most critical step while preparing a DNA biosensor is the immobilization of DNA probe on the surface of a sensing device such as an electrode. The amount of immobilized DNA probe will influence the accuracy, sensitivity, selectivity, and life of a DNA biosensor directly. Because of the high surface-to-volume ratio and excellent biological compatibility, nano-materials can enlarge the sensing surface area to increase the amount of immobilized DNA greatly, and the DNA mixed with nano-materials can keep its biological activity well.

### 2.1. Nanoparticles

**Figure 1.**
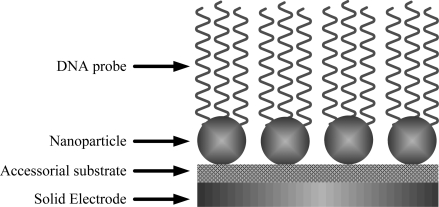
Schematic representation of nanoparticles as substrates for DNA attachment.

Over the past decade, the unique properties of nanoparticles have continued to attract considerable research attention. Nanoparticles, especially metal nanoparticles, offer excellent prospects for chemical and biological sensing because of their unique optical, electrical, and thermal properties as well as catalytic properties [17–19]. The nanoparticles were prepared mainly through reduction reactions in aqueous solutions containing the corresponding chloro-metallate anions, metal vapor synthesis routes, electrochemical depositions on inert bases, sol–gel, or deposition-precipitation [20–24]. The surface and geometry of nanoparticles can be tailored to bind a subset of biomarkers selectively. Figure 1 shows the schematic representation of nanoparticles as substrates for DNA attachment.

#### 2.1.1. Gold Nanoparticles

Gold (Au) nanoparticles are a hot study topic lately and they play a key role in DNA biosensors. Thiol–Au (SH–Au) linkages usually were used to bind Au nanoparticles covalently with solid electrodes or DNA because of the strong affinity of covalent bonds between sulfur atoms and gold atoms. Jin *et al*. [25] immobilized the calf thymus DNA on the surface of Au nanoparticles which were co-immobilized at a gold electrode through 4,4′-bis(methanethiol)biphenyl (MTP) molecules by an assembly process. Their results indicated that the Au nanoparticles modified gold electrode can enlarge the electrode surface area and enhance the amount of immobilized single stranded DNA (ssDNA) greatly, and the surface coverage value of DNA molecules decreased as the size of the gold nanoparticles increased. Fu *et al*. [26] also fabricated an Au nanoparticles modified DNA biosensor though thiol–Au linkages. The thiol groups of 3-mercaptopropyltrimethoxysilane (MPTS) served as binding sites for the covalent attachment of MPTS to a gold electrode surface. After hydrolysation and condensation, the polymerized monolayer, one-dimensional network of MPTS (1dMPTS) was combined together into a two-dimensional sol–gel network (2dMPTS). The second silane layer (B2dMPTS) was formed by immersing the electrode back into the MPTS solution, and then the Au nanoparticles were chemisorbed onto the thiol groups of the second silane layer. Finally, the mercapto oligonucleotide was self-assembled onto the surface via the Au nanoparticles. The results indicated that oligonucleotide immobilized in this way exhibits a good sensitivity, selectivity, stability and a long-term maintenance of bioactivity. Many other researchers [27–33] have used different reagents containing thiol groups to improve the DNA attachment though Au nanoparticles.

Au nanoparticles as substrates for DNA attachment though other linkages were also reported in several publications. Spadavecchia *et al*. [34] deposited the Au nanoparticles directly onto the surface of silicon (Si) and sapphire (Al_2_O_3_) substrates by a physical method to prepare a DNA biosensor. Pan [35] prepared a DNA biosensor by self-assembling horseradish peroxidase-linked single-stranded DNA (HRP-ssDNA) onto gold nanoparticles-modified composite membrane at a carbon paste electrode (CPE). Yang *et al*. [36] fabricated the Au nanoparticles by electropolymerizing 2,6-pyridine-dicarboxylic acid (PDC) on the glassy carbon electrode (GCE), and then DNA probe was immobilized on the Au nanoparticles by the interaction of Au with DNA. Kim *et al*. [37] deposited Au nanoparticles onto a porous anodic alumina (PAA) layer chip surface to form a “caplike” layer on the top of the oxide nanostructures in an orderly fashion.

#### 2.1.2. Platinum Nanoparticles

Platinum (Pt) nanoparticles were also used as substrates to improve DNA immobilization due to their high catalytic activities. Zhu *et al*. [38] fabricated an electrochemical DNA biosensor using Pt nanoparticles combined with multi-walled carbon nanotubes. Multi-walled carbon nanotubes and platinum nanoparticles were dispersed in Nafion, and then they were used to modify a glassy carbon electrode (GCE) surface. Oligonucleotides with amino groups at the 5′ end were covalently linked onto carboxylic groups of MWCNTs on the electrode. Due to the ability of carbon nanotubes to promote electron-transfer reactions and the high catalytic activity of platinum nanoparticles for chemical reaction, the sensitivity of presented electrochemical DNA biosensor was remarkably improved.

#### 2.1.3. Metal-Oxide Nanoparticles

Zirconia (ZrO_2_) is a thermally stability, chemically inert, non- toxicity inorganic oxide with affinity for the groups containing oxygen, and therefore it is an ideal candidate material for immobilization of biomolecules containing oxygen or phosphate groups. Thus, ZrO_2_ is an ideal material for immobilization of DNA. Zhu *et al*. [39] electrodynamically deposited ZrO_2_ thin films onto the bare gold electrode by cycling the potential between −1.1 and +0.7 V (versus Ag/AgCl) at a scan rate of 20 mV/s. Oligonucleotide probes with phosphate group at the 5′ end were attached onto the ZrO_2_ thin films. Yang *et al*. [36,40] also used ZrO_2_ coupled with carbon nanotubes to improve DNA attachment to the electrode.

Praseodymium oxide (Pr_6_O_11_) has many unique properties, such as a high dielectric constant, a large band gap, high electron affinity, and it is also relatively easy to present as a thin film. Shrestha *et al*. [41] developed a method for the chemical attachment of thiol-modified oligonucleotides on a Pr_6_O_11_ surface. Pr_6_O_11_ was deposited on a tin-doped indium oxide (ITO) surface to form an ultrathin layer with a larger internal surface area, and then the thiol-modified oligonucleotide attached to an amine-modified Pr_6_O_11_ surface.

#### 2.1.4. Silicon Nanoparticles

There are also a few reports concerning nonmetallic nanoparticles as substrates for DNA attachment, mainly focused on silicon nanoparticles. Zhang *et al*. [42] constructed a biocompatible and uniform interface based on silica nanoparticles derivatized with amino groups for the effective immobilization and sensitive sequence-specific detection of calf thymus DNA. Atomic force microscopy (AFM) and scanning electron microscopy (SEM) results showed that a monolayer of silica nanoparticles can be formed on a gold electrode using cysteine self-assembly monolayer as binder medium. Quantitative results demonstrated that enhanced immobilization of ssDNA up to 1.6 × 10^−8^ mol/cm^2^ could be achieved owing to the larger surface area and the special properties of silica nanoparticles. Wei and Wang [43] also prepared a ruthenium (Ru) doped silica nanoparticle modified indium tin oxide electrode by electrostatic self-assembly technique.

### 2.2. Nanotubes

**Figure 2.**
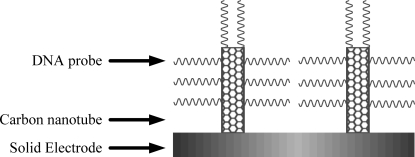
Schematic representation of CNTs as substrates for DNA attachment.

Carbon nanotubes (CNTs) were first noticed and characterized in 1991 by Iijima [44] of NEC Corporation in Japan. CNTs are a new allotrope of carbon originated from fullerene family, which can be described as a graphite sheet rolled up into a nanoscale-tube (which are single-wall carbon nanotubes, SWCNTs), or with additional grapheme tubes around the core of an SWCNT (which are multi-wall CNTs, MWCNTs) [45]. MWCNTs consist of two or more concentric cylindrical shells of graphene sheets coaxially arranged around a central hollow area with spacing between the layers which is close to that of the interlayer separation as in graphite (0.34 nm). In contrast, SWCNT are made of single grapheme (one layer of graphite) cylinders and have a very narrow size distribution (1–2 nm). Both types of nanotubes have the physical characteristics of solids and are microcrystals, although their diameters are close to molecular dimensions. CNTs offer unique electronic and mechanical properties combined with chemical stability. Combining a biological compound with CNT to monitor a biochemical event resulted in promising biosensors. Due to their catalytic effects, the DNA-end attachment via covalent linkage to the CNTs surface has been demonstrated (as shown in Figure 2) and further extended for the hybridization of the captured strands to their complementary target sequences. CNTs as sensing surfaces for nucleic acids detection have been extensively demonstrated.

#### 2.2.1. Direct Attachment of DNA to Functionalized CNTs

Tang *et al*. [46] modified the gold electrodes by carboxylic group-functionalized CNTs which activated using *N*-ethyl-*N*′-(3-dimethylaminopropyl) carbodiimide (EDC) and *N*-hydroxysuccinimido-biotin (NHS), then the synthesized ssDNA was covalently immobilized on the CNTs modified gold electrodes. Abdullin *et al*. [47] modified glassy-carbon electrodes (GCEs) with preoxidized CNTs. According to the data of atomic force microscopy, the layers of CNTs on GCEs possess a homogeneous nanostructurized surface. Guanine and deoxyguanosine monophosphate could be strongly adsorbed on GCE/CNT and oxidized at +690 and +930 mV (pH 7.0), respectively. Similar attachment of DNA onto CNTs was also reported by Kerman *et al*. [48], Erdem *et al*. [49] and Liang *et al*. [50].

#### 2.2.2. Indirect Attachment of DNA through Different Polymers

Polypyrrole (PPy) is a common material which acts as a bridge to connect DNA with CNTs. Xu *et al*. [51,52] reported the attachment of DNA to MWCNTs using PPy. The immobilization relied on the doping of nucleic acid probes within electropolymerized PPy film onto a carboxylic group-functionalized MWCNTs (MWNTs-COOH) modified electrode. Oligonucleotide probes served as the solo counter anions during the growth of conducting PPy film on the carbon nanotube modified electrode. Cai *et al*. [53] and Qi *et al*. [54] also reported DNA biosensors using electrodes assembled by CNTs and immobilizing DNA probe within PPy.

Chitosan is another material used as a bridge for DNA attachment to CNTs. Li *et al*. [55] fabricated a biosensor based on chitosan doped with CNTs. It was found that CNTs could enhance the electroactive surface area threefold (0.28 ± 0.03 and 0.093 ± 0.06 cm^2^ for chitosan-CNTs-modified electrodes and chitosan-modified electrodes, respectively). Bollo *et al*. [56] also dispersed CNTs in chitosan to modify glassy carbon electrodes (GCE), and obtained an approving result.

Other materials used for DNA attachment to CNTs were also reported. Guo *et al*. [57] described a method of DNA immobilization on CNTs through amidation. DNAs were covalently immobilized on MWCNTs via diimide-activated amidation between the carboxylic acid groups on the carbon nanotubes and the amino groups on DNA bases. Similarly, Jung *et al*. [58] covalently immobilized DNA oligonucleotides to prepatterned SWCNT multilayer films by amidation. Guo *et al*. [59] studied the electrostatic assembly of calf thymus DNA on MWCNTs modified gold electrode via poly (diallyldimethylammonium chloride) (PDDA), a cationic polyelectrolyte. They found that the electrode modified with MWCNTs had significantly enhanced the effective electrode surface area in addition to providing negatively charged groups for the electrostatic assembly of cationic polyelectrolyte.

### 2.3. Nanowires

Nanowires fabricated by polyaniline were utilized as substrates for DNA attachment in some literatures [60,61]. Nanowires of conducting polymers were directly synthesized through electrochemical deposition procedure in an aniline-containing electrolyte solution using the glassy carbon electrode (GCE) as the working electrode. The diameter of the nanowires ranged from 80 to 100 nm. Oligonucleotides with phosphate groups at the 5′ end were covalently linked onto the amino groups of polyaniline nanowires on the electrode in the presence of the water-soluble coupling reagent EDC.

Nebel *et al*. [62] introduced a novel biosensing platform by combination of a) geometrically controlled DNA bonding using vertically aligned diamond nano-wires and b) the superior electrochemical sensing properties of diamond as transducer material. Ultra-hard vertically aligned diamond nano-wires were electrochemically modified to bond phenyl linker-molecules to their tips which provided mesospacing for DNA molecules on the transducer. The nano-wires were generated by reactive ion etching of metallically boron doped atomically smooth single crystalline CVD diamond. Electro- and bio-chemical sensor properties reveal sensitivities of 2 pM on 3 mm^2^ sensor areas and superior DNA bonding stability over 30 hybridization/denaturation cycles.

### 2.4. Nanoclusters

Nanoclusters also reported as substrates for DNA attachment. Zhu *et al*. [63] synthesized a CdS nanocluster directly with free carboxyl groups on its surface in aqueous solution. Because of the free carboxyl groups on the nanocluster surface, the CdS nanocluster enables it to be easily bonded to amino-group capped oligonucleotides. The assay relied on the hybridization of the target DNA with the CdS nanocluster oligonucleotide DNA probe, followed by the dissolution of the CdS nanoclusters anchored on the hybrids and the indirect determination of the dissolved cadmium ions by sensitive anodic stripping voltammetry (ASV) at a mercury-coated glassy carbon electrode (GCE). The combination of the large number of cadmium ions released from each dsDNA hybrid with the remarkable sensitivity of the electrochemical stripping analysis for cadmium at mercury-film GCE allowed detection of the complementary sequence of DNA at levels as low as 0.2 pmol/L.

## 3. Nano-Materials as Signal Amplifier for Hybridization

When we detect small-molecule ligands of biomedical interest, ligand binding may not significantly perturb the biosensor interface. In this situation, signal amplification may be useful. Signal amplification by labeling the analyte with nano-materials has been reported for DNA biosensors in many literatures.

### 3.1. Nanoparticles

**Figure 3.**
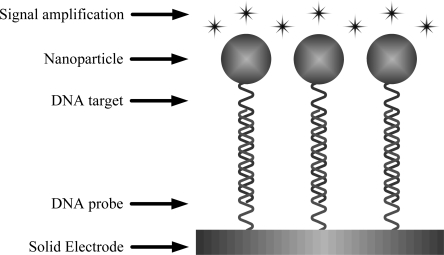
Schematic representation of nanoparticles as signal amplifiers for hybridization.

Figure 3 shows the schematic representation of nanoparticles as signal amplifier for hybridization. The materials of nanoparticles used as signal amplifier which reported in literatures include gold (Au), silver (Ag), cadmium sulfide (CdS). These nanoparticles are summarized for their use in DNA biosensors as follows.

#### 3.1.1. Gold Nanoparticles

Due to their electrochemical properties, Au nanoparticles have been used as signal amplifiers in many electrochemical DNA biosensors. Wang *et al*. [64] demonstrated that Au nanoparticles could amplify the electrochemical impedance and capacitance signals for the model fluorescein/antifluorescein system. Following immobilization of fluorescein onto Au through formation of a self-assembled monolayer, goat antifluorescein conjugated with 10 nm Au nanoparticles was introduced into the system. This resulted in an increase in the capacitance similar to 400 nF/cm^2^, whereas no change could be observed for goat antifluorescein without the Au nanoparticle conjugate. This allowed construction of high-sensitivity electrochemical impedance biosensors at a single low frequency, where the signal was sensitive to the interfacial R_ct_. This change in the electrochemical impedance signal of binding to goat antifluorescein conjugated with Au nanoparticles could be attributed to the much higher electrochemical activity of Au surfaces relative to the underlying organic layer. Amplification of voltammetric signal was also characterized by many researchers [65–67]. Li and Hu [68] developed an electrochemical determination method for analyzing sequence-specific DNA using ferrocene-capped gold nanoparticles/streptavidin conjugates. Thiolated DNA probes were covalently immobilized on a gold electrode with hexanethiol (HT) forming mixed self-assembled monolayer and hybridized with target DNA, containing a complementary sequence. Duplex (double-stranded) DNA formed on the gold surface. Then functional gold nanoparticles were introduced via strong interaction effect between biotin and streptavidin. The electrochemical signal of ferrocene covering on the gold nanoparticles enhanced obviously in cyclic voltammetry (CV) and differential pulse voltammetry (DPV).

Optical properties of Au nanoparticles were also used for optical DNA biosensor. Yao *et al*. [69] used Oligonucleotide (ODN)-capped Au nanoparticles in a sandwich assay of ODN or polynucleotide by a flow injection surface plasmon resonance (SPR). A carboxylated dextran film was immobilized onto the SPR sensor surface to eliminate nonspecific adsorption of ODN-capped Au-NPs. The tandem use of signal amplification via the adlayer of the ODN-capped Au-NPs and the differential signal detection by the bicell detector on the SPR resulted in a remarkable DNA detection level. Optical signal amplification by Au nanoparticles was also reported by Kalogianni *et al*. [70] and Martins *et al*. [71].

Au nanoparticles were also reported to be used as signal amplifiers for quartz crystal microbalance (QCM) biosensors [2,72–75]. Au nanoparticles were attached to the quartz crystal Au electrodes via different methods to increase the mass on the electrode surface, amplifying the signal of hybridization.

#### 3.1.2. Silver Nanoparticles

Ag nanoparticles have desirable compositions as oligonucleotides labels in electrochemical detection assays because Ag nanoparticles exhibit better electrochemical activity than Au ones. Cai *et al*. [76] reported an electrochemical DNA hybridization detection assay, using Ag nanoparticles as the oligonucleotide labeling tag. The assay relied on the hybridization of the target DNA with the Ag nanoparticle–oligonucleotide DNA probe, followed by the release of the Ag metal atoms anchored on the hybrids by oxidative metal dissolution. The result indicated that the electrochemical redox reaction of Ag carried out at a low potential, under 0.4 V, and it gave a well-defined sharp voltammetric peak. The oxidative peak of Ag colloid was approximately 100 times as large as that for Au colloid of the same size and amount. And Ag metal could be easily oxidized to the soluble ionic Ag^+^ with concentrated HNO_3_ then released from the hybrids, but complete dissolution of a gold tag required more severe conditions (1 M HBr containing 0.1 mM Br_2_) and the electrode might be damaged in this medium. Similar electrochemical characterizations of Ag nanoparticles used in DNA biosensors were done by Fu *et al*. [77] and K’Owino *et al*. [78].

#### 3.1.3. Cadmium Sulfide Nanoparticles

Cadmium sulfide (CdS) nano-material is a semiconductor material with attractive electrochemical properties. CdS as oligonucleotide labeling tags for detection of DNA hybridization have been reported by several researchers. Xu *et al*. [79] used a type of CdS nanoparticles covalently binding with the amine groups modified target ssDNA to enhance the electrochemical impedance spectroscopy (EIS) signal. Because of the negative charges, space resistance and semiconductor characteristics of CdS tags, the use of CdS nanoparticle greatly improved DNA hybridization detection sensitivity. Peng *et al*. [80] also used CdS nanoparticles to enhance the EIS signal. They described an ODN sensor based on electropolymerization of a conducting polymer (polypyrrole) in the presence of a sample containing ODN(s). The resulting trapped ODN(s) were then probed by addition of complimentary sequence ODN. By incorporating CdS nanoparticles with the probe, a significant improvement in sensor sensitivity was observed.

### 3.2. Nanotubes

The conductive properties of CNTs suggest that they could mediate electron transfer reactions and enhance the relative electrochemical reactivity with electroactive species in solution when used as the electrode material or substrates modified on solid electrodes. As electrode or substrates modified materials, CNTs show better electrochemical behavior than traditional glass carbon electrodes, carbon paste electrodes and any other carbon electrodes [81]. Therefore, part of the previous introduction of CNTs used as substrates for DNA attachment in electrochemical biosensors could also be regarded as electrochemical signal amplifier. The guanine oxidation signal of double stranded calf-thymus DNA after 3 min accumulation was 20 times higher at a CNTs modified glass carbon electrodes cross-linked with glutaraldehyde (GTA) than at a bare GCE using differential pulse voltammetry, while the peak potential was around 45 mV less positive. And the guanine oxidation signal demonstrated to be highly reproducible, with 3.4% RSD for five different electrodes [56]. Zhang *et al*. [82] compared the electrochemical behavior of K_3_Fe(CN)_6_ at a bare glassy carbon electrode and an electrochemically activated glassy carbon electrode which was treated in the absence or presence of multi-walled CNTs under identical experimental conditions. The results indicate that the electrochemical response of the K_3_Fe(CN)_6_ at the CNTs activated electrode was the strongest among these three kinds of electrodes.

The unique optical properties make CNTs ideal optical probes for tagging biomolecules. CNTs exhibit strong Raman signals as well as fluorescence emissions in the near infrared region. And such signals do not blink or photobleach under prolonged excitation, which is an advantage in optical nano-biomarker applications. The intense Raman scattering from CNTs provides a large amount of information about the structure and properties of nanotubes with some of the highest known cross-sections for single molecules. Hwang *et al*. [83] presented single-stranded DNA conjugated SWCNT probes to locate a particular sequence of DNA within a complex genome. Chromosomal DNAs of human fibroblasts and *Escherichia coli* were used as a target and a control, respectively. Southern blotting, which used photostable Raman signals of nanotubes instead of fluorescent dyes, demonstrated excellent sensitivity and specificity of the probes. Their results showed that SWCNTs may be used as generic nano-biomarkers for the precise detection of specific kinds of genes. Cao *et al*. [84] developed SWCNT-based molecular probes by conjugating single-stranded DNA (ssDNA) with SWCNT. The results showed that SWCNTs exhibited sharp absorption peak distributions in the UV–vis–NIR range when they were individually dispersed in aqueous solution medium. DNA hybridization on the sidewall of SWCNT resulted in systematic red shifts of the absorption spectra of semiconducting nanotubes. This research demonstrated that ssDNA–SWCNTs probes might be used to detect specific kinds of DNA oligonucleotides as optical nano-biosensors.

**Figure 4.**
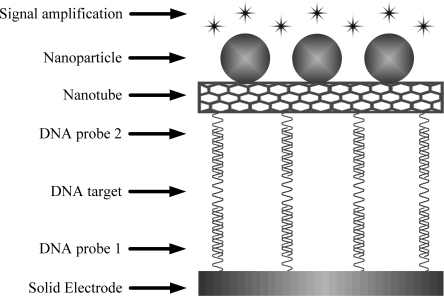
Schematic representation of nanotubes as signal amplifiers for hybridization.

Recently, CNTs have also been utilized as a novel support material to concentrate nanoparticles or enzyme molecules on it as a more powerful DNA hybridization indicator than using a single nanoparticle or enzyme molecule indicating DNA hybridization (as shown in Figure 4). Wang *et al*. [67] described an effective method for amplifying electrical detection of DNA hybridization based on CNTs carrying a large number of CdS particle tracers. Such use of CNT amplification platforms was combined with an ultrasensitive stripping voltammetric detection of the dissolved CdS tags following dual hybridization events of a sandwich assay on a streptavidin modified 96-well microplate. Anchoring of the monolayer-protected quantum dots to the acetone-activated CNT was accomplished via hydrophobic interactions. SEM images indicated that the nanocrystals were attached along the CNT sidewall, with a loading of around 500 particles per CNT. A substantial (∼500 fold) lowering of the detection limit was obtained compared to conventional single particle stripping hybridization assays, reflecting the CdS loading on the CNT carrier. A large excess (250 fold) of non-complimentary oligonucleotides had minimal effect on the response. They also demonstrated the use of CNTs for dramatically amplifying enzyme-based bioaffinity electrical sensing DNA [85]. In the new bioaffinity assays, CNTs played a dual amplification role in both the recognition and transduction events, namely as carriers for numerous enzyme tags and for accumulating the product of the enzymatic reaction. Such coupling of several CNT-derived amplification processes led to the low detection limit. Li *et al*. [86] developed an ultrasensitive electrogenerated chemilummescence (ECL) detection method of DNA hybridization based-on SWCNT carrying a large number of ruthenium complex tags. The probe of single strand DNA (ssDNA) and ruthenium complex were loaded at SWCNT, which was taken as an ECL probe. When the capture ssDNA with a thiol group was self-assembled onto the surface of gold electrode, and then hybridized with target ssDNA and further hybridized with the ECL probe to form DNA sandwich conjugate, a strong ECL response was electrochemically generated. Lee *et al*. [13] demonstrated a method of highly sensitive colorimetric detection of target DNA sequences amplified by the novel CNT-based label. Atomic force microscope (AFM) images confirmed that a monolayer of horseradish peroxidase and detection probe molecules was immobilized along the carboxylated CNT carrier. The resulting CNT labels significantly enhanced the nucleic acid assay sensitivity by at least 1000 times compared to that of conventional labels used in enzyme-linked oligosorbent assay (ELOSA).

### 3.3. Nanowires

Gao *et al*. [87] fabricated arrays of highly ordered silicon nanowires using complementary metal-oxide semiconductor compatible technology and investigated their applications in biosensors. Peptide nucleic acid (PNA) capture probe-functionalized silicon nanowires arrays showed a concentration-dependent resistance change upon hybridization to complementary target DNA that is linear over a large dynamic range with a detection limit of 10 fM. As with other silicon nanowires biosensing devices, the sensing mechanism can be understood in terms of the change in charge density at the silicon nanowires surface after hybridization, the so-called “field effect”. The silicon nanowires array biosensor discriminated satisfactorily against mismatched target DNA.

### 3.4. Nanoclusters

Hu *et al*. [88] developed a novel surface plasmon resonance (SPR) biosensor which used a co-sputtering method utilizing a multi-target sputtering system to fabricate the dielectric films (SiO_2_) with embedded Au nanoclusters. It was shown that the sensitivity of the SPR biosensor could be improved by adjusting the size and volume fraction of the embedded Au nanoclusters in order to control the surface plasmon effect. The DNA hybridization experimental results could achieve 10-fold improvement in the resolution performance compared with the conventional SPR biosensors.

## 4. Conclusions and Future Prospects

In conclusion, different nano-materials have been successfully applied to DNA biosensors as substrates for DNA attachment and as labels for signal amplification, specifically. Table 1 lists part of the recent reports on the use of nano-materials to improve DNA biosensors.

(1) When nano-materials are used as substrates for DNA attachment, the smaller size the nano-material is, the better result a DNA biosensor can provide. As the size of a crystal is reduced, the surface-to-volume ratio of a crystal is increased, thus it can enlarge the electrode surface area to greatly enhance the amount of immobilized DNA. But this conclusion is only applicable under the condition that the shape of nano-materials is regular, otherwise we cannot obtain a repeatable result. Similar conclusion holds true when nano-materials are used as signal amplifiers.

(2) Due to the diverse properties of different nano-materials, utilizing two or more types of nano-materials could enhance the good qualities as well as offset the insufficiency of each individual nano-material, which could produce better results than that using only one type of nano-materials. For example, Cai *et al*. [94] described an electrochemical detection method for analyzing sequence-specific DNA using gold nanoparticle DNA probes and subsequent signal amplification step by silver enhancement. The assay relied on the electrostatic adsorption of target oligonucleotides onto the sensing surface of the glassy carbon electrode (GCE) and its hybridization to the gold nanoparticle-labeled oligonucleotides DNA probe. After silver deposition onto gold nanoparticles, binding events between the probe and target were monitored by the differential pulse voltammetry (DPV) signal of the large number of silver atoms anchored on the hybrids at the electrode surface. Coupled with this ‘nanoparticle-promoted’ reduction of silver signal amplification method, the sensitivity of this electrochemical DNA biosensor was increased by approximately two orders of magnitude. Utilizing multiple nano-materials was also reported in some other literatures [95,96], including part of the aforementioned literatures.

(3) Although great efforts have been made in all DNA biosensor technologies in order to eliminate the role of the PCR from their protocols, this goal has not yet been achieved [97]. Many scientists have paid attention to the applications of DNA biosensors in the detection of DNA sequences in real samples, for example, GMO, hepatitis virus and so on [70,98,99], but almost all the protocols could not be achieved without PCR. As the development of nano-technology, nano-materials with smaller size and/or with improved biological and chemical properties would substantially enhance the accuracy, selectivity and sensitivity of DNA biosensors. With the rapid development of both nano-technology and biosensing technology, it can be envisaged that DNA biosensors without PCR amplification will come true in the near future.

## Figures and Tables

**Table 1.** Recent reports of nano-materials used in DNA biosensors.

**Author**	**Form**	**Material**	**Size**	**Detection Method**	**Purpose**	**Effect**	**Reference**

Baca, Zhou *et al*. 2004	Particle	Ferrocene-capped Au nanoparticle	10 nm	Voltammetry	Signal amplification	The detection level for glutathione was 1 nM	[65]
Bollo, Ferreyra *et al*. 2007	Tube	MWCNT	1–5 μm long and (30 ± 15) nm diameter	Voltammetry	Substrates for DNA attachment	The guanine oxidation signal of double stranded DNA after 3-min accumulation was 20 times higher than at bare GCE	[56]
Cao, Kim *et al*. 2008	Tube	SWCNT	Diameter: 0.7–1.1 nm, average length: 1 μm	UV–VIS–NIR absorption spectra	Substrates for DNA attachment	DNA hybridization on the sidewall of SWCNT resulted in systematic red shifts of the absorption spectra of semiconducting nanotubes	[84]
Chen, Wu *et al*. 2008	Particle	Au	Average diameter: 20 nm	Circulating-flow QCM	Signal amplification	Effectively amplified the signals in frequency change	[2]
Fu, Yuan *et al*. 2005	Particle	Au	16 nm	EIS	Substrates for DNA attachment	Detection limit: 5.0 × 10^−9^ M	[26]
Gao, Agarwal *et al*. 2007	wire	Silicon	50–80 nm	Electrical measurements	Substrates for DNA attachment	Detection limit: 10 fM	[87]
Ghanbari, Bathaie *et al*. 2008	Wire	Polypyrrole	30–90nm in diameter	SEM & EIS & Voltammetry & FTIR spectroscopy	Substrates for DNA attachment	A binding constant value of 4.08 × 10^5^ ± 0.05M^−1^ was obtained.	[89]
Hu, Chen *et al*. 2004	Cluster	Au & SiO_2_	4.0 nm	SPR	Signal amplification	10-fold improvement in the resolution performance	[88]
Kalogianni, Koraki *et al*. 2006	Particle	Au		Dry-reagent visual detection	Signal amplification	Detection limit: 0.16 nM	[70]
Kang, Li *et al*. 2007	Particle	Au		Voltammetry & EIS	Substrates for DNA attachment	detection limit could reach the concentration of 10 × 10^−9^ M	[27]
Kerman, Morita *et al*. 2004	Tube	MWCNT	10–20 nm i.d. and 1–5 μm length	Square-wave voltammetry & UV-visible spectrophotometer	Substrates for DNA attachment	Detection limit: 10 pg/mL	[90]
Lee, Ye *et al*. 2007	Tube	SWCNT		AFM & Colorimetric detection	Substrates for HRP-DP	Enhanced sensitivity by at least 1000 times, and detection limit: 1 × 10^−12^ M	[13]
Li, Liu *et al*. 2005	Tube	MWCNT		CV & UV–visible spectrophotometric	Substrates for DNA attachment	Enhance the electroactive surface area threefold	[55]
Lu, Lin *et al*. 2007	Hollow ball	Au	3.5 nm	AFM & QCM	Substrates for DNA attachment	Detected limit is extend from 10^−9^ to 10^−12^ M	[30]
Nebel, Yang *et al*. 2008	Wire	Diamond	10 nm diameter	AFM & SEM & EIS & Voltammetry	Substrates for DNA attachment	The sensitivity of 2 pM on 3 mm^2^ sensor areas and superior DNA bonding stability over 30 hybridization/denaturation cycles.	[62]
Peng, Soeller *et al*. 2006	Particle	CdS	Average diameter: 10 nm	EIS & Voltammetry	Signal amplification	Increase in electron-transfer resistance per CdS–ODN bound is 500 times larger than the increase per unlabeled ODN-probe bound.	[80]
Qi, Li *et al*. 2007	Tube	MWCNT		SEM & DPV	Substrates for DNA attachment	Detection limit: 8.5 × 10^−11^ M	[54]
Sun, Choy *et al*. 2009	Particle	Ag		QCM & EIS	Substrates for DNA attachment	Enhancement of 3.3 times for binding of complementary DNA has been shown	[3]
Tsai, Chang *et al*. 2006	Particle	Au		FESEM & Voltammetry	Substrates for DNA attachment	The number density of the AuNP spots in the multi-AuNPs biosensor is much higher than that in the single-AuNPs biosensor	[32]
Wang, Liu *et al*. 2003	Tube & Particle	SWCNT & CdS	5–10 nm diameter & 2 μm length	SEM & Stripping-voltammetric	SWCNT used as substrates for CdS tags	Detection limit: 40 pg/mL	[91]
Xia, Chen *et al*. 2008	Particle	CdS	About 600 nm	CV	Substrates for DNA attachment	Detection range: 1 × 10^−1^ to 1 × 10^−5^ μM	[92]
Xu, Cai *et al*. 2004	Particle	CdS	Average diameter: 5 nm	EIS	Signal amplification	Sensitivity is improved to two orders of magnitude compared with non-CdS tagged DNA sequences	[79]
Xu, Jiang *et al*. 2004	Tube	MWCNT	30–50 nm diameter & 1–10 μm length	EIS & Voltammetry	Substrates for DNA attachment	Detection limit: 5 × 10^−11^ M	[51]
Yang, Wang *et al*. 2007	Tube & Particle	MWCNT & ZrO_2_		CV	Substrates for DNA attachment	Detection limit: 7.5 × 10^−11^ M	[40]
Yao, Li *et al*. 2006	Particle	Au		SPR	Signal amplification	A 39-mer target at a quantity as low as 2.1 × 10^−20^ mol, corresponding to 1.38 fM in a 15 μL solution, can be measured.	[69]
Zhang, Chen *et al*. 2004	Particle	SiO_2_		AFM & SEM & X-ray & photoelectron spectroscopy & EIS & Voltammetry	Substrates for DNA attachment	Enhanced immobilization of ss-DNA up to 1.6 × 10^−8^ mol/cm^2^, markedly larger than the commonly accepted saturation monolayer adsorption (2.77 × 10^−10^ mol/cm^2^)	[42]
Zhang, Wang *et al*. 2007	Tube	MWCNT	30 nm diameter	AFM & Voltammetry	Substrates for DNA attachment	Detection limit of 7.5 nM for guanine and 150 ng/mL for acid denatured DNA	[82]
Zhang, Yang *et al*. 2009	Tube & shuttle	SWCNT & CeO_2_		SEM & EIS & Voltammetry	Substrates for DNA attachment	The dynamic range for detecting the sequence specific DNA was from 1.0 × 10^−12^ mol/L to 1.0 × 10^−7^ mol/L, and the detection limit was 2.3 × 10^−13^ mol/L	[93]
Zhu, Chang *et al*. 2005	Tube & Particle	MWCNT & Pt	40–60 nm diameter & 1–10 μm length	CV & DPV	Substrates for DNA attachment	Detection limit: 1.0 × 10^−11^ M	[38]
